# Residual Axillary Metastases in Node-Positive Breast Cancer Patients After Neoadjuvant Treatment: A Register-Based Study

**DOI:** 10.1245/s10434-024-15354-1

**Published:** 2024-05-04

**Authors:** Frederikke Munck, Maj-Britt Jensen, Ilse Vejborg, Maria K. Gerlach, Maja V. Maraldo, Niels T. Kroman, Tove H. F. Tvedskov

**Affiliations:** 1 Department of Breast Surgery, Herlev-Gentofte Hospital, Hellerup, Denmark; 2 Danish Breast Cancer Group, Copenhagen University Hospital – Rigshospitalet, Copenhagen, Denmark; 3 Department of Breast Examinations and Capital Mammography Screening, Herlev-Gentofte Hospital, Hellerup, Denmark; 4 Department of Pathology, Herlev-Gentofte Hospital, Hellerup, Denmark; 5 Department of Clinical Oncology, Center of Cancer and Organ Diseases, Copenhagen University Hospital – Rigshospitalet, Copenhagen, Denmark; 6 Danish Cancer Society, Copenhagen, Denmark; 7 Department of Breast Surgery, Herlev-Gentofte Hospital, Hellerup, Denmark

**Keywords:** Breast cancer, Axillary staging, Targeted axillary dissection, Axillary metastases, Neoadjuvant chemotherapy for breast cancer

## Abstract

**Background:**

Lymph node (LN) metastasis after neoadjuvant chemotherapy (NACT) generally warrants axillary lymph node dissection, which opposes guidelines of upfront surgery in many cases. We investigated the risk of having additional metastases in the axilla when the LNs removed by targeted axillary dissection (TAD) harbored metastases after NACT. We aimed to identify subgroups suitable for de-escalated axillary treatment.

**Methods:**

This register-based study used data from the Danish Breast Cancer Cooperative Group database. Data were analyzed with logistic regression models. The primary outcome was the metastatic burden in non-TAD LNs in patients with positive TAD LNs after NACT.

**Results:**

Among 383 patients, < 66.6% positive TAD LNs (adjusted odds ratio [OR] 0.34, 95% confidence interval [CI] 0.17–0.62), only isolated tumor cells (ITCs) [OR 0.11, 95% CI < 0.01–0.82], and breast pathological complete response (pCR) [OR 0.07, 95% CI < 0.01–0.56] were associated with a low risk of having more than three positive non-TAD LNs. In 315 patients with fewer than three positive non-TAD LNs, the proportion of positive TAD LNs (OR 0.45, 95% CI 0.27–0.76 for 33.3–66.6% vs. > 66.6%), size of the TAD LN metastasis (OR 0.14, 95% CI 0.04–0.54 for ITC vs. macrometastasis), tumor size at diagnosis (OR 0.30, 95% CI 0.15–0.64 for 20–49 mm vs. ≥ 50 mm) and breast pCR (OR 0.38, 95% CI 0.15–0.96) were associated with residual LN metastases in the axilla.

**Conclusions:**

Breast pCR or ITC only in TAD LNs can, with reasonable certainty, preclude more than three positive non-TAD LNs. Additionally, patients with only ITCs in the TAD LN had a low risk of having any non-TAD LN metastases after NACT. De-escalated axillary treatment may be considered in both subgroups.

Balancing axillary staging and local control against the risk of chronic morbidity is essential when performing axillary staging after neoadjuvant chemotherapy (NACT), especially considering axillary pathological complete response (axillary pCR) rates of 40–60%.^1–4^

No consensus exists on the optimal axillary staging method after NACT when patients with breast cancer are clinically node-positive at diagnosis.^5^ Some centers use sentinel lymph node biopsy (SLNB), emphasizing the removal of at least three sentinel nodes (SNs) or the use of a dual tracer technique, while others perform targeted axillary dissection (TAD).^5^ With TAD, the positive lymph node (LN) is marked before NACT and excised along with the SN at surgery.

When SN or TAD LN display pCR after NACT, omission of axillary lymph node dissection (ALND) is considered safe as the false negative rate is low. The false negative rate is 2–4% when performing TAD^4,6,7^ and 5–9% for SLNB, when a dual tracer is used^8^ or at least three SNs can be excised for evaluation.^9,10^ In the case of SN metastases after NACT, the rate of non-SN metastases ranges between 47 and 71%^11–13^ irrespective of macro- or micrometastases in the SN.^12,14^ Furthermore, speculations regarding relative chemoresistance in post-NACT tumor cell deposits compared with chemotherapy-naïve metastases can also hinder the acceptance of de-escalated axillary treatment strategies.^15^

Therefore, guidelines recommend ALND for node-positive patients after NACT regardless of the size or extent of metastasis found at SLNB or TAD.^16,17^ This approach opposes primary surgery, where non-SN metastasis rates in patients with small SN metastases [isolated tumor cells (ITC) and micrometastases] are 9–20%^18,19^ and where ALND is often no longer recommended when patients are treated with breast-conserving surgery and macrometastases are found in 1–2 SNs.^16,20^

To our knowledge, the residual metastatic load in axillary LNs in case of metastases in TAD LNs after NACT has not been investigated. This represents a knowledge gap in de-escalating axillary surgery. Perhaps patient and treatment factors could aid in identifying subgroups of patients with metastases in the TAD LN after NACT but with low metastatic burden in the non-TAD LNs. These subgroups may be offered de-escalated treatment options instead of ALND, thereby avoiding the high risks of pain, paresthesia, and lymphedema associated with ALND.^21,22^

In this study, we investigated the clinicopathological factors associated with a low metastatic burden in the axilla in node-positive breast cancer patients staged with TAD after NACT. The study aimed to aid in selecting patients suitable for de-escalated axillary treatment despite TAD LN metastases after NACT. We also describe the axillary pCR rate.

## Methods

### Data Acquisition

This was a retrospective cohort study including all Danish breast cancer patients with histologically confirmed node-positive disease diagnosed from 1 January 2016 to 31 August 2021. Data were retrieved from the Danish Breast Cancer Cooperative Group (DBCG) database, which includes nearly complete data on diagnosis and treatment of all Danish breast cancer patients.^23^

The data contained information on date of birth, date of breast biopsy and surgery, type of surgery, type of axillary surgery, number of LNs (axillary LNs, SNs, and marked LNs for TAD) removed with and without metastases, metastasis size, breast tumor histology and receptor subtype, breast tumor size at diagnosis and upon histopathological evaluation of the surgical specimen, malignancy grade and type of neoadjuvant treatment administered. Missing information on malignancy grade or tumor receptor subtype from the biopsy was retrieved from the pathology reports of the surgical specimen. Ethnicity data are generally not collected in Danish registers and patient files and are therefore not reported.

Axillary pCR was defined as absence of residual tumor in the removed LNs. Estrogen receptor (ER) status was considered positive when ≥ 1% of tumor cells were ER-positive. The tumor was considered HER2+ if the immunohistochemical (IHC) score was 3+ or if a HER2+ tumor had either a HER2/CEP17 ratio ≥ 2.0, with an average HER2 copy number ≥ 4.0, or a HER2/CEP17 ratio < 2.0, with an average HER2 copy number ≥ 6.0.^24^ LN metastases were classified according to the American Joint Committee on Cancer (AJCC) 8th edition staging manual.^25^ The node-positive status at diagnosis was histologically verified in all patients using fine-needle aspiration cytology or core-needle biopsy.

After data extraction, validation was performed to ensure that patients treated with TAD were identified. For this purpose, pathology reports and information from patients’ medical files were used.

Patients were excluded if they had inflammatory breast cancer, former ipsilateral breast cancer, fewer than four or more than eight cycles of neoadjuvant treatment, < 14 weeks between biopsy and surgery, or if the NACT regimen differed from the recommended guideline. No marking of a metastatic LN at diagnosis and no attempt at SLNB after NACT were also excluded, as this was regarded as no attempt at TAD. Patients in whom the marked LN was not found at surgery were also excluded.

### Neoadjuvant Treatment, Surgery, and Adjuvant Radiotherapy

Since March 2021, the recommended NACT regimen in the DBCG guideline has been eight cycles of anthracycline- and taxane-based treatment.^26^ Before March 2021, the recommended NACT regimen consisted of six cycles of anthracycline- and taxane-based treatment.^27^ The DBCG guidelines included dual blockade with trastuzumab and pertuzumab in HER2+ patients receiving NACT in early 2016.^27^

In the DBCG guidelines, NACT patients with node-positive disease at diagnosis are recommended adjuvant radiotherapy with a radiation field that includes the axilla if ALND is not performed.^28^ The DBCG guidelines also recommend that patients with node-positive disease after NACT receive ALND regardless of the extent of metastatic deposit.^29^ In the DBCG surgical guidelines, a dual tracer is recommended for SLNB.^17^

### Outcomes and Statistical Analysis

The primary outcome was residual metastatic burden in non-TAD LNs in patients with metastases in TAD LNs after NACT. A high metastatic burden in the non-TAD LNs was set to more than three non-TAD LN metastases, while a low metastatic burden was set at one to three non-TAD LN metastases. These cut-off values were similar to those defined in the Dutch MARI protocol.^30^ The secondary outcome was the axillary pCR rate stratified by the receptor subtype of the tumor.

The primary outcome was analyzed in a subset of data that included patients who underwent ALND due to metastases in the TAD LNs. The association between clinicopathological characteristics and high versus no/low metastatic burden in non-TAD LNs was analyzed using the Firth logistic regression model. Variables that were statistically significant in the univariate tests were included in the multivariate analysis and backward stepwise selection was used for the final model. For ordinal categorical variables, levels were pooled if there were no statistically detectable differences between them. The distribution of number and proportion of positive TAD LNs was examined according to the primary outcome in a three-way table before the regression model was created. This was performed to examine whether the variables were correlated. The reported odds ratios (ORs) and confidence intervals (CIs) were calculated using the profiled penalized log-likelihood method.

In the subgroup that included only patients with three or fewer non-TAD LN metastases, associations between clinicopathological characteristics and low versus no residual metastatic burden in non-TAD LNs were analyzed by logistic regression. The multivariate logistic regression model included variables that were statistically significant in univariate analysis. The level of significance was set to 0.05. A Hosmer–Lemeshow goodness-of-fit test was used for both regression models with satisfactory output.

The predicted and observed probabilities stratified by the associated factors for each model were assessed and estimates were reported with corresponding Clopper–Pearson CIs.

All statistical analyses were calculated using R statistical software (R Core Team, 2021, Vienna, Austria, with packages ‘logistf’, ‘epitools’, ‘PropCIs’, and ‘readxl’).^31^

The Legal Department of the Capital Region of Denmark (j.no. P-2019-811), Danish Patient Safety Authority, and Center for Regional Development of the Capital Region (j.no. 31-1521-208) approved this project. Permission for data retrieval from the DBCG database was approved by the DBCG Surgical Board and the DBCG Steering Board.

## Results

**Fig. 1:**
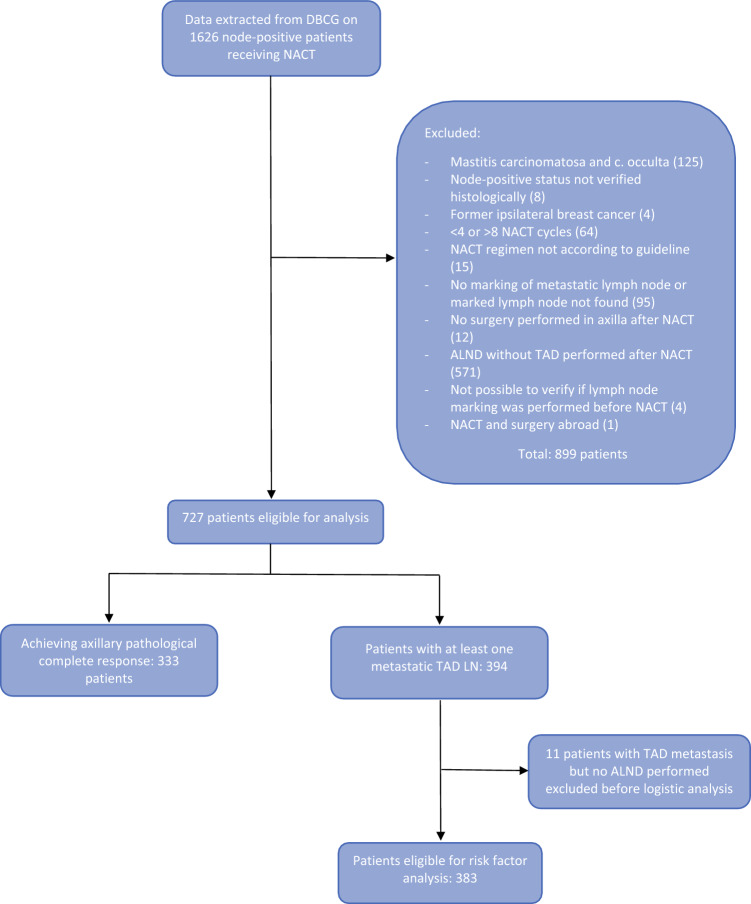
Included node-positive breast cancer patients receiving NACT and staged by TAD. *NACT* neoadjuvant chemotherapy, *TAD* targeted axillary dissection, *DBCG* Danish Breast Cancer Cooperative Group, *ALND* axillary lymph node dissection, *LN* lymph node

A total of 1626 patients were identified in the DBCG database. After excluding 899 patients, the study data included 727 patients eligible for analysis (see Fig. 1 for details on the excluded patients). Overall, 333 patients had axillary pCR in the TAD LNs, corresponding to 46% (333/727), and these patients were not included in further analyses because ALND was not performed. According to the receptor status of the breast tumor, the axillary pCR rate was 13% (34/260) for ER+/HER2− tumors, 56% (86/153) for ER−/HER2− tumors, 82% (106/130) for ER^−^/HER2*+* tumors, and 58% (107/184) for ER+/HER2*+* tumors.

The remaining 394 patients had at least one positive TAD LN after NACT. Of these, 383 underwent ALND and were included in the analysis of residual axillary metastases. The distribution of clinical characteristics according to non-TAD LN metastasis burden is shown in Table 1.

**Table 1:** Clinical characteristics in 383 Danish breast cancer patients receiving NACT and staged by TAD with subsequent ALND due to residual lymph node metastatic burden treated from 2016 to 2021

	Total (*N*)	No positive non-TAD LNs [*n* (%)]	One to three positive non-TAD LNs [*n* (%)]	More than three positive non-TAD LNs [*n* (%)]
Total	383	188 (49)	127 (33)	68 (18)
Age, years				
23–49	158	78 (49)	57 (36)	23 (15)
50–81	225	110 (49)	70 (31)	45 (20)
Tumor size at diagnosis (ultrasound), mm				
< 20	57	28 (49)	24 (42)	5 (9)
20–49	266	140 (53)	75 (28)	51 (19)
≥ 50	56	18 (32)	27 (48)	11 (20)
NA		2	1	1
Surgery type				
Mastectomy	171	79 (46)	58 (34)	34 (20)
Breast-conserving surgery	212	109 (51)	69 (33)	34 (16)
Malignancy grade				
1	29	12 (41)	11 (38)	6 (21)
2	262	125 (48)	89 (34)	48 (18)
3	92	51 (55)	27 (29)	14 (15)
Histological subtype				
Invasive ductal carcinoma	365	180 (49)	119 (33)	66 (18)
Invasive lobular carcinoma	10	4 (40)	5 (50)	1 (10)
Other	8	4 (50)	3 (38)	1 (13)
Receptor subtype				
ER−/HER2−	55	26 (47)	17 (31)	12 (22)
ER−/HER2+	22	12 (55)	6 (27)	4 (18)
ER+/HER2−	229	101 (44)	81 (35)	47 (21)
ER+/HER2+	77	49 (64)	23 (30)	5 (6)
Proportion of positive TAD LNs				
< 33.3%	21	14 (67)	6 (29)	1 (5)
33.3–66.6%	135	86 (64)	36 (27)	13 (10)
> 66.6%	227	88 (39)	85 (37)	54 (24)
Number of positive TAD nodes				
1	219	116 (53)	74 (34)	29 (13)
2	104	51 (49)	36 (35)	17 (16)
3	38	16 (42)	12 (32)	10 (26)
> 3	22	5 (23)	5 (23)	12 (55)
TAD metastasis size				
Isolated tumor cells	27	24 (89)	3 (11)	0 (0)
Micrometastasis	41	23 (56)	14 (34)	4 (10)
Macrometastasis	315	141 (45)	110 (35)	64 (20)
Invasive carcinoma at surgery				
Breast non-pCR	348	160 (46)	120 (35)	68 (20)
Breast pCR	35	28 (80)	7 (20)	0 (0)

*ALND* axillary lymph node dissection, *LNs* lymph nodes, *NACT* neoadjuvant chemotherapy, *TAD* targeted axillary dissection, *ER* estrogen receptor, *HER2* human epidermal growth factor receptor 2, *pCR* pathological complete response, *NA* not available

### Risk of High Metastatic Burden in the Non-targeted Axillary Dissection (TAD) Lymph Nodes

Among the 383 patients with at least one positive TAD LN after NACT and ALND, the odds of having more than three positive non-TAD LNs were significantly associated with an ER+/HER2+ receptor subtype, a proportion of positive TAD LNs < 66.6%, ITC in the positive TAD LN, and breast pCR (Table 2).

**Table 2:** Univariate and multivariate logistic regression analysis to determine the presence of more than three non-TAD metastatic LNs in Danish breast cancer patients staged with TAD after NACT with subsequent ALND

	Unadjusted analysis (*n* = 383)		Adjusted analysis (*n* = 379)		After backward stepwise elimination (*n* = 379)	
	OR (95% CI)	*p* value	OR (95% CI)	*p* value	OR (95% CI)	*p* value
Age, years		0.17				
23–49	0.69 (0.39–1.18)					
50–81	1.00					
Tumor size at diagnosis, mm		0.054				
< 20	0.44 (0.16–1.01)					
≥ 20	1.00					
Malignancy grade		0.49				
1 + 2	1.00					
3	0.80 (0.41–1.48)					
Receptor subtype		0.02		0.17		
ER+/HER2−	1.00		1.00			
ER−/HER2−	1.10 (0.53–2.19)		1.63 (0.75–3.41)			
ER−/HER2+	0.93 (0.28–2.55)		1.41 (0.40–4.30)			
ER+/HER2+	0.29 (0.10–0.68)		0.43 (0.15–1.03)			
Proportion of positive TAD LNs		< 0.001		< 0.001		< 0.001
< 66.6%	0.32 (0.17–0.59)		0.35 (0.18–0.64)		0.34 (0.17–0.62)	
66.6–100%	1.00		1.00		1.00	
TAD metastasis size		0.005		0.062		0.02
Isolated tumor cells	0.07 (< 0.01–0.52)		0.15 (< 0.01–1.17)		0.11 (< 0.01–0.82)	
Micrometastasis	0.47 (0.15–1.17)		0.75 (0.23–2.05)		0.68 (0.21–1.80)	
Macrometastasis	1.00		1.00		1.00	
Breast carcinoma at surgery		< 0.001		0.01		< 0.01
Breast pCR	0.06 (< 0.01–0.42)		0.06 (< 0.01–0.49)		0.07 (< 0.01–0.56)	
Breast non-pCR	1.00		1.00		1.00	

*TAD* targeted axillary dissection, *LNs* lymph nodes, *ALND* axillary lymph node dissection, *NACT* neoadjuvant chemotherapy, *ER* estrogen receptor, *HER2* human epidermal growth factor receptor 2, *pCR* pathological complete response, *OR* odds ratio, *CI* confidence interval

In the multivariate logistic regression analysis, the characteristics that had a statistically significant association with metastatic burden in the LNs were the proportion of positive TAD LNs (OR 0.34, 95% CI 0.17–0.62, *p* < 0.001, for ≤ 66.6% vs. > 66.6%), TAD metastasis size (OR 0.11, 95% CI < 0.01–0.82, *p* = 0.02, for ITC vs. macrometastases), and breast pCR (OR 0.07, 95% CI < 0.01–0.56, *p* < 0.01).

In the risk model (Table 3), 54 patients (54/383, 14%) who had either breast pCR or ITC in the TAD LN had a low risk of having more than three non-TAD metastases, with zero observed cases (pooled 95% CI < 0.01–6.6) and predicted risk ≤ 4%. Conversely, the risk model identified 86% (329/383) of patients as the high-risk group. In the high-risk group, 68 patients had more than three positive non-TAD LNs. As a diagnostic test, the sensitivity and specificity of the model were 0.17 and 1, respectively.

**Table 3:** Risk of having more than three non-TAD metastases among 383 breast cancer patients receiving TAD after NACT according to protective factors

Identified protective factors			Model predicted probabilities, %	Dataset, c/n	Pooled risk, % (95% CI)
Breast pCR	TAD metastasis size	TAD LN-positive proportion, %			
Yes	Isolated tumor cells	0–66.6	0.1	0/5	0.0 (< 0.01–6.6)
		> 66.6	0.3	0/3	
	Micrometastases	0–66.6	0.6	0/4	
		> 66.6	1.9	0/7	
	Macrometastases	0–66.6	1.0	0/6	
		> 66.6	2.8	0/10	
No	Isolated tumor cells	0–66.6	1.4	0/11	
		> 66.6	4.0	0/8	
	Micrometastases	0–66.6	8.2	1/15	6.7 (1.7–31.9)
		> 66.6	20.9	3/15	20.0 (4.3–48.1)
	Macrometastases	0–66.6	11.6	13/115	11.3 (6.1–18.6)
		> 66.6	27.9	51/184	27.7 (21.4–34.8)

*TAD* targeted axillary dissection, *NACT* neoadjuvant chemotherapy, *pCR* pathological complete response, *LN* lymph node, *CI* confidence interval, *c/n* cases/total number

### Low Non-TAD Metastatic Burden versus No Non-TAD Metastases

After excluding patients with more than three metastatic non-TAD LNs, 315 patients remained for low (1–3) versus no non-TAD metastasis analysis. In these 315 patients, the odds of having low non-TAD LN metastatic burden were significantly associated with tumor size at diagnosis, proportion of positive TAD LNs, size of TAD metastasis, and breast pCR (Table 4).

**Table 4:** Univariate and multivariate logistic regression analysis to determine the presence of one to three non-TAD metastatic lymph nodes in Danish breast cancer patients with three or fewer non-TAD metastases found by ALND after TAD treated from 2016 to 2021

	Unadjusted analysis (*n* = 315)		Adjusted analysis (*n* = 312)	
	OR (95% CI)	*p* value	OR (95% CI)	*p* value
Age, years		0.56		
23–49	1.15 (0.73–1.80)			
50–81	1.00			
Tumor size at diagnosis, mm		< 0.01		< 0.01
< 20	0.57 (0.25–1.28)		0.46 (0.19–1.13)	
20–49	0.36 (0.18–0.69)		0.30 (0.15–0.64)	
≥ 50	1.00		1.00	
Malignancy grade		0.41		
1	1.28 (0.54–3.02)			
2	1.00			
3	0.74 (0.43–1.27)			
Receptor subtype		0.28		
ER+/HER2−	1.00			
ER−/HER2−	0.82 (0.41–1.61)			
ER−/HER2+	0.62 (0.22–1.73)			
ER+/HER2+	0.59 (0.33–1.04)			
Proportion of positive TAD LNs		< 0.01		< 0.01
< 33.3%	0.44 (0.16–1.21)		0.58 (0.20–1.69)	
33.3–66.6%	0.43 (0.27–0.71)		0.45 (0.27–0.76)	
> 66.6%	1.00		1.00	
Type of TAD metastasis		< 0.01		< 0.01
Isolated tumor cells	0.16 (0.05–0.55)		0.14 (0.04–0.54)	
Micrometastasis	0.78 (0.38–1.59)		0.99 (0.45–2.19)	
Macrometastasis	1.00		1.00	
Breast carcinoma at surgery		0.02		0.03
Breast pCR	0.33 (0.14–0.79)		0.38 (0.15–0.96)	
Breast non-pCR	1.00		1.00	

*TAD* targeted axillary dissection, *ALND* axillary lymph node dissection, *ER* estrogen receptor, *HER2* human epidermal growth factor receptor 2, *LNs* lymph nodes, *pCR* pathological complete response, *OR* odds ratio, *CI* confidence interval, *c/n* cases/total number

In the multivariate logistic regression analysis, the clinicopathological characteristics that remained statistically significantly associated with non-TAD LN metastatic burden were the proportion of positive TAD LNs (OR 0.45, 95% CI 0.27–0.76, *p* < 0.01 for 33.3–66.6% vs. > 66.6%), TAD LN metastasis size (OR 0.14, 95% CI 0.04–0.54, *p* < 0.01 for ITC vs. macrometastases), breast tumor size (OR 0.30, 95% CI 0.15–0.64, *p* < 0.01 for 20–49 mm vs. ≥ 50 mm), and breast pCR (OR 0.38, 95% CI 0.15–0.96, *p* = 0.03).

The analysis further showed that the odds for low non-TAD LN metastatic burden were decreased for the smallest breast tumor size (OR 0.46, 95% CI 0.19–1.13 for < 20 mm vs. ≥ 50 mm) and the lowest proportion of positive TAD LNs (OR 0.58, 95% CI 0.20–1.69 for < 33.3% vs. > 66.6%), but the CI did not reach statistical significance for these levels.

In the risk model (Table 5), 9% (27/312) of the patients were in the low-risk group and had an 11.1% (95% CI 2.9–29.2) observed risk of having one to three non-TAD metastases. The remaining 91% (285/312) of patients were in the high-risk group. When the model was regarded as a diagnostic test, the sensitivity and specificity were 0.13 and 0.98, respectively.

**Table 5:** Risk of having any non-TAD metastases among 312 breast cancer patients with fewer than three positive non-TAD lymph nodes after NACT

Identified protective factors				Model predicted probabilities, %	Dataset, c/n	Pooled risk, % (95% CI)
TAD metastasis size	Tumor size at diagnosis, mm	Breast pCR	TAD LN positive proportion, %			
Isolated tumor cells	< 50	Yes	0–66.6	2.5	1/4	11.1 (2.9–29.2)
			> 66.6	5.3	0/2	
		No	0–66.6	6.4	0/8	
			> 66.6	12.8	2/5	
	≥ 50	Yes	0–66.6	7.2	0/1	
			> 66.6	14.4	0/1	
		No	0–66.6	17.0	0/3	
			> 66.6	30.6	0/3	
Micro- and macrometastases	< 50	Yes	0–66.6	14.8	1/9	11.1 (0.3–48.2)
			> 66.6	27.3	5/16	31.3 (11.0–58.7)
		No	0–66.6	31.4	32/103	31.1 (22.3–40.9)
			> 66.6	49.7	58/120	48.3 (39.1–57.6)
	≥ 50	Yes	0–66.6	34.4	0/1	NR
			> 66.6	53.0	0/1	NR
		No	0–66.6	57.9	7/10	70.0 (34.8–93.3)
			> 66.6	74.8	20/25	80.0 (59.3–93.2)

*TAD* targeted axillary dissection, *NACT* neoadjuvant chemotherapy, *NR* not reported, *pCR* pathological complete response, *LN* lymph node

## Discussion

In this register-based study, we examined clinicopathological factors associated with high (more than three), low (one to three), or no residual metastatic burden in the non-TAD LNs found by ALND in patients with metastases in the TAD LNs after NACT. These results suggest that achieving breast pCR or having only ITC in the TAD LN as the largest metastatic deposit can rule out more than three metastatic non-TAD LNs. With a low risk of more than three non-TAD LN metastases, patients with these clinicopathological characteristics may be considered for de-escalation to axillary radiotherapy instead of ALND.

In addition, ITC in the TAD LN as the largest metastasis was associated with an 11% risk of one to three non-TAD metastases in a subanalysis of patients with three or fewer non-TAD metastases. Along with metastasis size and breast pCR, the results showed that a smaller tumor at diagnosis and a lower proportion of positive TAD LNs were also associated with a decreased risk of having one to three non-TAD LN metastases. However, some variable levels of these parameters did not reach statistical significance, perhaps because of the small number of observations with these characteristics.

We developed two models for predicting more than three positive non-TAD LNs and no further positive non-TAD LNs in patients with three or fewer positive non-TAD LNs. The specificity of the models was high, indicating safe de-escalation of surgical axillary treatment. However, the sensitivity of both models was low, indicating that considerable overtreatment of the axilla would be present despite using the models.

In our study, 46% of patients achieved axillary pCR after NACT. These results are consistent with reported rates, ranging between 27 and 57%.^1–3,32–36^ As the axillary pCR rate varies with receptor subtype, patient selection for NACT and inclusion in studies may explain the variation between studies.

With a high chance of having no positive non-TAD LNs after NACT, the patient may achieve axillary staging and local control with TAD alone. However, the uncertainty in the risk estimates in this subgroup warrants caution if decisions on no further axillary treatment are based thereon. Therefore, the correct cut-off point for the risk of non-TAD metastases can discussed. In primary surgery, studies have reported that the risk of non-SN metastases in patients with ITC and micrometastases is 9–20%.^18,19,37^ These patients are no longer offered ALND. Accordingly, a cut-off value with a 9–20% risk of non-TAD LN metastases could be suggested. Nonetheless, the clinical significance of residual chemoresistant metastases left in the axilla after NACT might differ from that of primary surgery, possibly necessitating a lower risk cut-off.

When considering eligibility for axillary de-escalation, it should also be noted that the LN metastatic burden is a surrogate endpoint for regional recurrence. To date, evidence on TAD has primarily focused on its feasibility and false-negative rates. While the reported false-negative rate of TAD is 2–4%,^4,6,7^ and thereby no higher than the false-negative rate of approximately 4–9% of SLNB in primary surgery,^38,39^ only sparse data exist regarding regional recurrence after TAD. The few existing regional recurrence studies indicate a low risk at 3 years of follow-up. The MARI study reported that 98% of their cohort was free of axillary recurrences and the SenTa study reported an axillary recurrence rate of 1.4%.^30,40^

To date, prior studies have used two approaches to select subgroups of NACT patients with a predicted low risk of non-TAD LN metastases. One approach has been to investigate the clinicopathological characteristics that predict axillary pCR in patients receiving direct ALND after NACT. With this design, studies have found that breast pCR,^2,32–34^ a higher degree of breast tumor response clinically or on imaging,^3,32,35,36^ tumor receptor subtype,^2,3,32,34–36^ and a smaller LN diameter or no palpable lymphadenopathy after NACT^3,32,35^ are associated with the possibility of axillary pCR. The studies differ regarding obtaining histological verification of node-positive status at diagnosis and whether all HER2+ patients received HER2-targeted therapy.^35,36^

An alternative approach to identifying patients with limited axillary metastatic burden is to determine the risk factors for non-SN metastases in the presence of positive SNs. Studies have reported an association between SN extracapsular extension,^13,41^ lymphovascular invasion,^41–43^ a higher proportion or count of positive SNs,^11,13,14,41,44^ older age,^13^ tumor receptor subtype,^14,44,45^ size of the SN metastasis,^14,41,44^ breast pCR,^11,45^ tumor size,^41^ and presence of multicentric or multifocal disease.^14,43^ However, regarding the association between tumor receptor subtypes and non-SN metastases, the reported results are conflicting.^42,43^ Additionally, clinically node-positive and node-negative patients are often included alike;^13,42–44^ thus, estimates and results cannot be directly compared with those of TAD patients.

In contrast to these previous studies, we only included patients with histologically verified LN metastases who underwent axillary staging by TAD, making the results valid in a clinical setting. To our knowledge, this is the first study to investigate the residual metastatic burden in axillary LNs after NACT in a consecutively treated cohort of breast cancer patients staged by TAD. This study’s register-based design and use of DBCG data allowed for a large dataset of consecutive patients and a high degree of completeness.^23^

The study limitations include the risk of misclassification bias in register-based studies and restriction of results to the available data. Second, if data from the biopsy were missing, we registered the histological variables from the surgical specimen, knowing that an NACT-induced change in some parameters could occur. Third, although we had access to data on NACT cycles per patient, and the effect of NACT cycles on the outcome would add interesting knowledge, we chose not to include this in our analysis. We omitted this information because, with our data source, it was unfeasible to distinguish between patients allocated to six cycles of NACT and those allocated to eight cycles and terminated early due to toxicity. A guideline amendment during the study period also caused a treatment shift towards more patients allocated to eight cycles. Fourth, patients excluded for receiving fewer than four NACT cycles may have been terminated early because their response to NACT was poor. Fifth, while we had access to data on the number and proportion of positive TAD LNs, only one of these parameters could be included in the models, as these variables were highly interdependent. Finally, exclusion criteria were set to ensure the study's internal validity, resulting in a high exclusion rate.

## Conclusion

ITCs in TAD LNs or breast pCR only were associated with low odds of having more than three positive non-TAD LNs in the axilla. These patients could be considered for de-escalation from ALND to axillary radiotherapy. Considering the patients with ITCs in the TAD LN alone, few seemed to have any positive non-TAD LNs after NACT, and no further surgical axillary treatment could be considered. It is hoped these results will contribute to the selection of subgroups of patients whose axillary metastatic burden is sufficiently low to allow de-escalation of axillary treatment after NACT.
